# Surgical repair of paraesophageal hernia resolves unexplained iron deficiency anemia in the vast majority of patients: a propensity-matched multicenter study

**DOI:** 10.1007/s00464-026-12900-8

**Published:** 2026-05-21

**Authors:** Fahim Kanani, Maria Shaiban, Chaya Shwaartz, Majd Khalil, Katia Dayan, Rula Francis, Daniel Solomon, Yonatan Lessing, Narmin Zoabi, Eviatar Kuhnreich, Eviatar Nesher, Amir Szold, Boaz Sagi, Nir Messer

**Affiliations:** 1 Department of Surgery, Asuta Medical Centres, Tel Aviv, Israel; 2 Department of Surgery, Wolfson Medical Centre, Tel Aviv University Faculty of Medicine, Tel Aviv, Israel; 3 Department of Surgery, Tel Aviv Sourasky Medical Centre, Tel Aviv University Faculty of Medicine, Tel Aviv, Israel; 4 Department of Transplantation, Beilenson Medical Center, Petah Tiqua, Israel; 5 Department of Transplantation and HPB Surgery, Toronto General Hospital, Toronto, Canada; 6 Department of Surgery, Asuta Medical Centre Ashdod, Ben-Gurion University Faculty of Health Sciences, Beer-Sheva, Israel; 7 Department of Gastroenterology, Sheba Medical Centre, Tel Aviv University Faculty of Medicine, Tel Aviv, Israel; 8 Department of Transplantation Surgery, Beilinson Medical Center (Rabin Campus), Clalit Health Services, Gray Faculty of Medicine and Health Sciences, Tel Aviv University, Tel Aviv, Israel

**Keywords:** Paraesophageal hernia, Iron deficiency anemia, Hiatal hernia repair, Propensity score matching, Laparoscopic surgery

## Abstract

**Background:**

Paraesophageal hernias (PEH) frequently associate with iron deficiency anemia (IDA) through unclear mechanisms. We investigated whether surgical repair effectively treats unexplained IDA in patients with PEH.

**Methods:**

This multicenter retrospective study (2010–2024) analyzed 1700 patients undergoing hiatal hernia repair across Asuta Medical Centers. After excluding patients with identifiable bleeding sources (Cameron lesions, erosive gastritis, ulcers), 522 patients remained: 136 with unexplained IDA and 386 controls. Propensity score matching (1:3) aligned 99 anemic patients with 297 controls based on age, sex, BMI, and comorbidities. Primary endpoint was anemia resolution at 12 months (hemoglobin > 12 g/dL females, > 13 g/dL males without iron supplementation).

**Results:**

Baseline hemoglobin was 10.5 ± 1.4 vs 13.9 ± 1.2 g/dL (*p* < 0.001). Anemia severity was mild (62.6%), moderate (31.3%), or severe (6.1%). Despite profound anemia, 42.4% were completely asymptomatic at presentation. Prior iron therapy had failed in 78.2% over 5.8 ± 3.2 months. Anemia resolution occurred in 93.4% (92/99) at 12 months, with 88.9% achieving normal hemoglobin by 3 months. Mean hemoglobin increased by 3.2 g/dL (95% CI 2.9–3.5, *p* < 0.001), with ferritin normalizing from 19.3 ± 11.8 to 95.3 ± 38.5 ng/mL. Iron supplementation was successfully discontinued in 82.8% of patients. Among 16 patients with hernia recurrence, 75% maintained normal hemoglobin despite anatomical failure. Control group hemoglobin remained unchanged (13.9 ± 1.2 to 13.9 ± 1.1 g/dL, *p* = 0.892). Due to only 7 non-resolution events, multivariate analysis was not performed; however, diabetes (71.4% vs. 28.3%) and chronic kidney disease (28.6% vs. 3.3%) were associated with persistent anemia.

**Conclusions:**

Unexplained IDA resolves in > 90% of patients following PEH repair, with durable results often maintained despite anatomical recurrence. These findings suggest PEH repair should be considered for patients with unexplained, refractory IDA. Prospective studies are needed to establish evidence-based criteria for this underrecognized indication.

**Supplementary Information:**

The online version contains supplementary material available at 10.1007/s00464-026-12900-8.

Paraesophageal hernias (PEH) account for approximately 5–10% of all hiatal hernias and encompass a broad clinical spectrum, ranging from incidental radiographic findings to acute, life-threatening complications requiring emergent surgical intervention [1, 2]. Among their less overt but clinically significant manifestations is iron deficiency anemia (IDA), reported in 30–50% of patients with PEH across multiple case series [3, 4]. While well recognized, this association remains insufficiently understood and often underappreciated in routine surgical practice. The prevailing hypothesis attributes PEH-associated anemia to Cameron lesions. However, reported detection rates of Cameron lesions vary widely (8.5–32%) and are frequently absent in anemic patients, suggesting that additional or alternative mechanisms may be involved [5, 6]. Proposed contributors include chronic venous congestion of herniated stomach, intermittent arterial insufficiency, repetitive diaphragmatic shear stress, altered gastric pH affecting iron bioavailability, and nutritional compromise related to dysphagia [7–9].

The clinical presentation of PEH-associated anemia often defies conventional expectations. Unlike typical gastrointestinal bleeding sources, many patients present without dysphagia, reflux symptoms, or overt bleeding manifestations. This “silent” anemia frequently leads to delayed diagnosis, with patients undergoing extensive hematologic workups before the anatomical culprit is identified [10, 11].

Although the association between PEH and IDA is well established, the therapeutic impact of surgical repair on anemia has been addressed in only a limited number of studies. These limited investigations are further constrained by methodological heterogeneity, inconsistent definitions of hematologic response, and variable follow-up durations [12–15].

In this context, we conducted a propensity score-matched cohort study to evaluate the impact of paraesophageal hernia repair on anemia resolution in patients with IDA. Our secondary aim was to identify preoperative factors associated with hematologic response following surgical intervention.

## Methods

This multicenter retrospective cohort study included all patients who underwent paraesophageal hernia (PEH) repair at Asuta Medical Centers between January 2010 and December 2024. Institutional review board approval was obtained (IRB# ASMC-0088-24).

Eligible patients were adults (≥ 18 years) with radiographically confirmed type II–IV PEH and unexplained iron deficiency anemia (IDA) diagnosed preoperatively, with a minimum of 12 months postoperative follow-up. Patients were excluded if they had undergone prior bariatric surgery; demonstrated potential bleeding sources on endoscopy (e.g., Cameron lesions, erosive gastritis, peptic ulcer disease) [16–18]; had hematologic disorders (e.g., thalassemia, hemoglobinopathies), active malignancy, dialysis-dependent renal disease, or severe malnutrition (defined as albumin < 2.5 g/dL or BMI < 18.5 kg/m^2^). Patients whose primary indication for surgical referral was documented failure of iron supplementation therapy—rather than hernia-related symptoms, anatomical progression, or complications—were excluded, in order to isolate a cohort in which PEH repair was performed on standard surgical grounds.

Patients were included if they had undergone a standardized preoperative evaluation, including upper endoscopy to exclude mucosal pathology and anatomical confirmation of paraesophageal hernia via a full upper gastrointestinal (UGI) contrast series and/or computed tomography of the chest and abdomen, with the modality selected based on clinical presentation and referring physician preference. The UGI contrast series was performed using barium sulfate suspension as the contrast agent, in accordance with standardized institutional radiology protocols across the Asuta Medical Center network; water-soluble iodinated contrast was not used in this elective cohort. The UGI contrast series served as the primary tool for hernia classification when CT alone did not provide adequate anatomical detail of the gastroesophageal junction position or degree of intrathoracic gastric migration; in selected patients, both modalities were utilized in accordance with established diagnostic algorithms for PEH evaluation [13]

Hernias were classified according to established criteria as type II (pure paraesophageal), type III (mixed sliding and paraesophageal with > 50% intrathoracic stomach), or type IV (herniation of additional abdominal viscera) [13]. Hernia size was quantified as the maximum crural defect diameter (cm) on axial CT imaging and by the estimated percentage of intrathoracic stomach displacement, as reported in the formal radiologic assessment. Hernia classification was based on the formal radiologic report generated at the time of each study by a board-certified radiologist; no independent re-reading of imaging was performed by the surgical team for research purposes.

Surgical repair was offered to patients with symptomatic presentation (dysphagia, regurgitation, or postprandial chest pain), evidence of gastric volvulus or history of obstruction, respiratory complications, or radiographic progression in medically fit individuals, consistent with current guideline recommendations [13]. In the IDA cohort, patients who were otherwise asymptomatic from a foregut standpoint were offered surgical repair when IDA meeting all inclusion criteria was the presenting indication and no alternative etiology had been identified following comprehensive workup, in accordance with the institutional protocol at Asuta Medical Centers. This approach reflects an expanding body of evidence supporting PEH repair as a definitive treatment for hernia-associated IDA even in the absence of classic mechanical symptoms.

All included patients underwent comprehensive anemia evaluation, including detailed bleeding history, colonoscopy (age > 50 or positive fecal occult blood test), gynecologic assessment (in reproductive-age women), and nutritional screening. All assessments were completed within 3 months prior to surgery. Patients with incomplete workup documentation were excluded. Iron deficiency anemia was defined according to modified WHO criteria [14]:

- Hemoglobin < 13 g/dL (males) or < 12 g/dL (females),
- Ferritin < 30 ng/mL (or < 100 ng/mL with CRP > 5 mg/L), and
- Supporting criteria when available: MCV < 80 fL, transferrin saturation < 20%

Patients lacking ferritin measurements or not meeting both primary criteria were excluded. The analysis was restricted to iron deficiency anemia, as it represents the most prevalent anemia subtype associated with PEH and its presumed mechanistic relevance. Patients with alternative or mixed anemia etiologies were excluded to ensure pathophysiologic homogeneity.

Patients were stratified into two cohorts based on preoperative hematologic parameters: an “IDA” cohort meeting all diagnostic criteria, and a “control” cohort composed of non-anemic patients undergoing PEH repair during the study period. Comprehensive baseline data were collected for all patients, including demographics, comorbidities, American Society of Anesthesiologists (ASA) physical status classification, medication use, symptomatology, and detailed hernia characteristics. Postoperative follow-up was conducted at 30 days (± 2 weeks), 3 months, 6 months, and 12 months, with additional long-term evaluation at 5 years when data were available. Hemoglobin, ferritin, and MCV were measured at each visit. Quality-of-life assessments included the Mini Nutritional Assessment (MNA) and the GERD–Health-Related Quality-of-Life (GERD-HRQL) [19] questionnaire. For patients with prospectively collected scores documented in the medical record, preoperative and 12-month values were extracted. For those lacking complete documentation, structured telephone interviews were conducted between March and May 2024. Potential recall bias was acknowledged.

Primary outcome was the hemoglobin level at 12 months postoperatively. Anemia resolution was defined as achievement of hemoglobin levels above WHO thresholds, documented on two separate occasions at least three months apart, without interval iron supplementation. Secondary outcomes included 30-day postoperative outcomes such as complications (graded by the Clavien–Dindo classification), transfusion requirements, and mortality; longer-term outcomes including hemoglobin trajectory at 6 months, 12 months, and 5 years (when available), anatomical recurrence, and changes in quality-of-life scores; and anemia-specific endpoints such as discontinuation of iron therapy and the relationship between hernia recurrence and sustained hematologic response.

Statistical analysis utilized R Studio Version 4.5.0. We performed 1:3 propensity score matching between IDA and control cohorts using logistic regression incorporating age, sex, BMI, ASA score, comorbidities, and hernia characteristics. Matching employed nearest-neighbor methodology with 0.2 standard deviation caliper width. Balance was confirmed using standardized mean differences < 0.1. Analyses included paired t-tests for hemoglobin comparisons, chi-square tests for categorical variables, and multivariable regression for predictors of anemia resolution. Significance was set at *p* < 0.05. Patients who received perioperative blood transfusion (*n* = 3 IDA, *n* = 2 controls) were retained in all analyses. Hemoglobin values recorded at routine postoperative follow-up were used for outcome ascertainment regardless of transfusion status, as transfusions were administered for intraoperative hemostatic management and not as treatment for IDA.

## Results

**Fig. 1:**
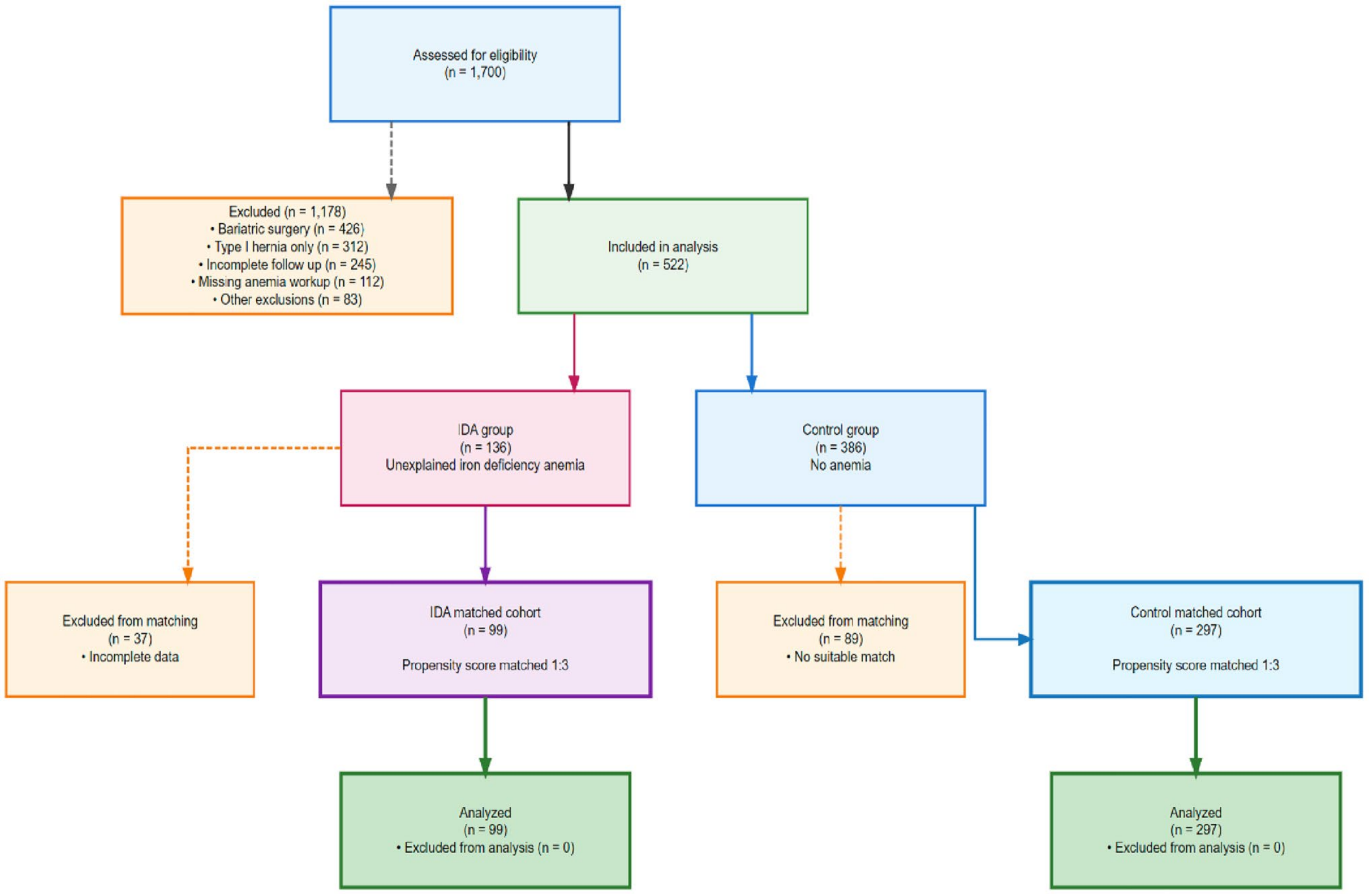
CONSORT flow diagram of study inclusion and exclusion

Between January 2010 and December 2024, 1700 patients underwent paraesophageal hernia repair across Asuta Medical Centers. Of these, 522 patients (30.7%) met inclusion criteria, including 136 (26.1%) with unexplained IDA and 386 (73.9%) non-anemic controls. Propensity score matching in a 1:3 ratio yielded 99 IDA patients matched to 297 controls; 37 patients with IDA were excluded due to lack of suitable matches (Fig. 1).

**Fig. 2:**
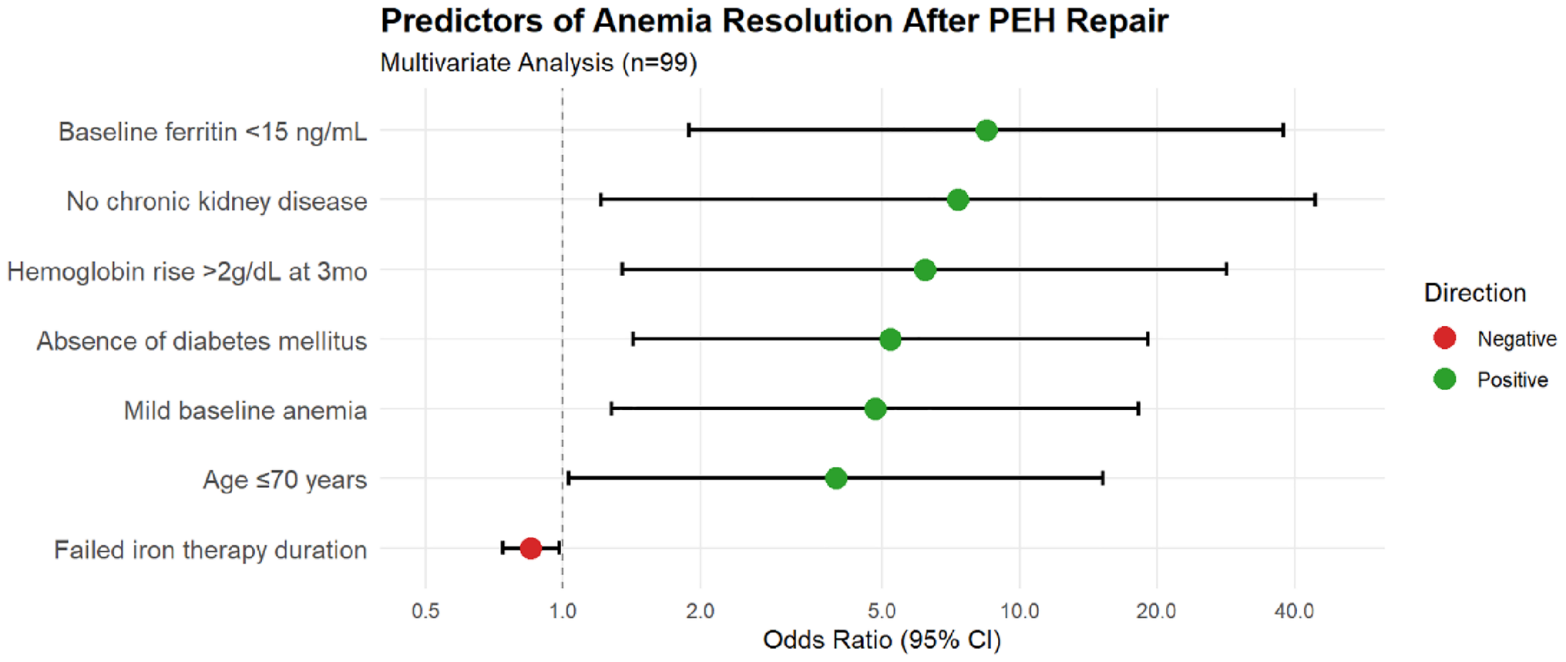
Predictors of anemia resolution after PEH repair—descriptive analysis

Following propensity score matching, baseline demographics were well balanced between cohorts (Table 1). Mean age was 61.9 ± 14.0 years in the IDA group versus 61.5 ± 14.1 years in controls (SMD = 0.027, *p* = 0.819). Female patients comprised 74 (74.7%) of the IDA group and 215 (72.4%) of controls (SMD = 0.053, *p* = 0.744). Body mass index averaged 24.0 ± 3.8 kg/m^2^ in IDA patients versus 24.2 ± 4.0 kg/m^2^ in controls (SMD = 0.046, *p* = 0.693). Diabetes mellitus was present in 31 patients (31.3%) with IDA and 101 controls (34.0%) (SMD = 0.057, *p* = 0.712). Chronic kidney disease affected 5 IDA patients (5.1%) versus 3 controls (1.0%) (SMD = 0.236, *p* = 0.024) (Fig. 2).

**Table 1:** Baseline demographics and comorbidities after propensity score matching

Variable	Total cohort (*n* = 396)	Anemic group (*n* = 99)	Control group (*n* = 297)	SMD	*P* value
Demographics					
Age, years	61.6 ± 14.1	61.9 ± 14.0	61.5 ± 14.1	0.027	0.819
Female sex, * n* (%)	289 (73.0)	74 (74.7)	215 (72.4)	0.053	0.744
BMI, kg/m^2^	24.1 ± 4.0	24.0 ± 3.8	24.2 ± 4.0	0.046	0.693
ASA score, * n* (%)				0.072	0.106
ASA 1	77 (19.4)	17 (17.2)	60 (20.2)		
ASA 2	264 (66.7)	66 (66.7)	198 (66.7)		
ASA 3	55 (13.9)	16 (16.2)	39 (13.1)		
Comorbidities, *n* (%)					
Diabetes mellitus	132 (33.3)	31 (31.3)	101 (34.0)	0.057	0.712
Hypertension	94 (23.7)	28 (28.3)	66 (22.2)	0.140	0.275
Hyperlipidaemia	52 (13.1)	15 (15.2)	37 (12.5)	0.078	0.606
Ischemic heart disease	16 (4.0)	7 (7.1)	9 (3.0)	0.185	0.141
COPD	10 (2.5)	3 (3.0)	7 (2.4)	0.042	1.000
Chronic kidney disease	8 (2.0)	5 (5.1)	3 (1.0)	0.236	0.024
Medications, *n* (%)					
PPI use	235 (59.3)	52 (52.5)	183 (61.6)	0.184	0.140
Aspirin use	52 (13.1)	17 (17.2)	35 (11.8)	0.153	0.233
Anticoagulation use	20 (5.1)	8 (8.1)	12 (4.0)	0.169	0.174
Follow-up^a^					
12 months, *n* (%)	357 (90.2)	89 (89.9)	268 (90.2)	–	0.95
5 years, *n* (%)	255 (64.4)	70 (70.7)	185 (62.3)	–	0.18

*Note* After 1:3 propensity score matching, 37 anemic patients were excluded due to lack of suitable matches, resulting in excellent covariate balance (all SMDs < 0.2 for matched variables)

*SMD* Standardized mean difference, *ASA* American Society of Anesthesiologists

^a^*Follow-up* Follow-up was not limited to surgical clinic visits. Patients were considered followed if any clinical, radiologic, or institutional medical record documented their status at 12 months and/or 5 years after surgery. Asymptomatic patients without surgical follow-up were included if no recurrence or reintervention was recorded

The IDA cohort demonstrated significantly lower baseline hemoglobin (10.5 ± 1.4 vs. 13.9 ± 1.1 g/dL, *p* < 0.001), MCV (77.9 ± 9.1 vs. 89.5 ± 6.2 fL, *p* < 0.001), and ferritin levels (19.3 ± 11.8 vs. 108.7 ± 41.2 ng/mL, *p* < 0.001) compared with controls. Anemia severity was classified as mild (Hb 10–11.9 g/dL) in females and (Hb 10–11.9 g/dL) in males showing 62 patients (62.6%), moderate (Hb 8–9.9 g/dL) in 31 patients (31.3%), and severe (Hb < 8 g/dL) in 6 patients (6.1%). Oral iron therapy was ongoing in 87 IDA patients (87.9%) for a mean duration of 5.8 ± 3.2 months, with 68 patients (78.2%) demonstrating inadequate response. Anemia-related symptoms were present in 34 patients (34.3%) versus 5 controls (1.7%, *p* < 0.001), (Table 2).

**Table 2:** Anemia characteristics and hematologic parameters

Variable	Anemic group (*n* = 99)	Control group (*n* = 297)	*P* value
Hematologic values			
Hemoglobin, g/dL	10.5 ± 1.4	13.9 ± 1.1	< 0.001
MCV, fL	77.9 ± 9.1	89.5 ± 6.2	< 0.001
Ferritin, ng/mL^a^	19.3 ± 11.8	108.7 ± 41.2	< 0.001
Transferrin saturation, %^b^	14.2 ± 6.3	28.5 ± 8.1	< 0.001
RDW, %	16.8 ± 2.3	13.2 ± 1.1	< 0.001
CRP, mg/L^c^	8.2 ± 6.1	3.1 ± 2.4	< 0.001
Albumin, g/dL	4.1 ± 0.5	4.2 ± 0.4	0.082
Anemia severity distribution			
Mild (Hb 10–11.9 g/dL)[F] & Hb 10–12.9 g/dL)[M]^e^	62 (62.6)	NA	–
Moderate (Hb 8–9.9 g/dL)	31 (31.3)	NA	–
Severe (Hb < 8 g/dL)	6 (6.1)	NA	–
Iron therapy details			
Receiving oral iron, *n* (%)	87 (87.9)	0 (0)	< 0.001
Duration of iron therapy, months	5.8 ± 3.2	NA	–
Inadequate response to iron, *n* (%)^d^	68 (78.2)	NA	–
IV iron attempted, *n* (%)	12 (12.1)	0 (0)	< 0.001
Anemia-related symptoms, *n* (%)			
Any anemia symptom	34 (34.3)	5 (1.7)	< 0.001
Fatigue	27 (27.3)	4 (1.3)	< 0.001
Dyspnea on exertion	15 (15.2)	2 (0.7)	< 0.001
Dizziness	8 (8.1)	0 (0)	< 0.001
Palpitations	6 (6.1)	1 (0.3)	0.002
Restless legs	9 (9.1)	3 (1.0)	0.001
Pica/Ice craving	4 (4.0)	0 (0)	0.003

^a^Available in 218 patients

^b^Available in 186 patients

^c^Available in 254 patients

^d^Of those on iron therapy

^e^Mild anemia defined per WHO sex-specific criteria: Hb 10.0–12.9 g/dL in males, 10.0–11.9 g/dL in females. The mild category includes 12 male patients with Hb 12.0–12.9 g/dL (mean 12.4 g/dL, range 12.0–12.9) who met all primary IDA diagnostic criteria and are included in the full cohort analysis

### Clinical presentation

Asymptomatic presentation was observed in 42 IDA patients (42.4%) versus 90 controls (30.3%, *p* = 0.036). Retrosternal pain was reported by 2 IDA patients (2.0%) versus 116 controls (39.1%, *p* < 0.001). Mean hernia size was 5.22 ± 0.76 cm in IDA patients versus 5.26 ± 0.69 cm in controls (*p* = 0.603). Cameron lesions, gastric ulcers, and erosive gastritis were absent in all IDA patients by study design but present in 52 (17.5%), 38 (12.8%), and 15 (5.1%) controls, respectively, (Table 3).

**Table 3:** Clinical presentation and hernia characteristics

Variable	Anemic group (*n* = 99)	Control group (*n* = 297)	*P* value
Presenting symptoms, *n* (%)			
Asymptomatic	42 (42.4)	90 (30.3)	0.036
Dysphagia	27 (27.3)	88 (29.6)	0.749
Retrosternal pain	2 (2.0)	116 (39.1)	< 0.001
Regurgitation	31 (31.3)	92 (31.0)	1.000
Early satiety	18 (18.2)	42 (14.1)	0.424
Weight loss > 5 kg	19 (19.2)	31 (10.4)	0.041
Melena/hematemesis	8 (8.1)	13 (4.4)	0.244
Recurrent pneumonia	4 (4.0)	4 (1.3)	0.216
Hernia characteristics			
Hernia size, cm	5.22 ± 0.76	5.26 ± 0.69	0.603
Hernia size > 6 cm, *n* (%)	32 (32.3)	85 (28.6)	0.576
Type II, *n* (%)	45 (45.5%)	140 (47.1%)	0.72
Type III, *n* (%)	50 (50.5%)	149 (50.2%)	0.85
Type IV, *n* (%)	4 (4.0)	8 (2.7)	0.35
Primary hernia, *n* (%)	94 (94.9)	293 (98.7)	0.080
Endoscopic findings, *n* (%)			
Cameron lesions	0 (0)^a^	52 (17.5)	< 0.001
Gastric ulcer	0 (0)^a^	38 (12.8)	0.001
Erosive gastritis	0 (0)^a^	15 (5.1)	0.141
Non-erosive gastritis	19 (19.2)	65 (21.9)	0.670
Barrett's esophagus	10 (10.1)	32 (10.8)	1.000
Esophagitis	21 (21.2)	55 (18.5)	0.658
Atrophic gastritis	3 (3.0)	7 (2.4)	0.985

^a^Excluded by study design (patients with visible bleeding sources were excluded from anemia cohort)

### Surgical outcomes

Operative time averaged 101.2 ± 54.9 min in IDA patients versus 99.9 ± 56.4 min in controls (*p* = 0.839). Intraoperative bleeding occurred in 10 IDA patients (10.1%) versus 8 controls (2.7%, *p* = 0.005). Major complications (Clavien–Dindo ≥ III) occurred in 9 IDA patients (9.1%) versus 31 controls (10.4%, *p* = 0.847). Blood transfusion was required in 3 IDA patients (3.0%) versus 2 controls (0.7%, *p* = 0.111). No 30-day mortality occurred in either group, (Table 4).

**Table 4:** Surgical outcomes and postoperative complications

Variable	Anemic Group (*n* = 99)	Control Group (*n* = 297)	*P* value
Operative details			
Surgery duration, min	101.2 ± 54.9	99.9 ± 56.4	0.839
Laparoscopic approach, *n* (%)	97 (98.0)	292 (98.3)	1.000
Mesh reinforcement, *n* (%)	96 (97.0)	289 (97.3)	1.000
Fundoplication type, *n* (%)			0.892
Nissen 360°	68 (68.7)	209 (70.4)	
Toupet 270°	21 (21.2)	58 (19.5)	
Dor 180°	8 (8.1)	24 (8.1)	
Thal 90°	2 (2.0)	6 (2.0)	
Gastropexy performed, *n* (%)	73 (73.7)	218 (73.4)	1.000
Intraoperative bleeding	10 (10.1)	8 (2.7)	0.005
Conversion to open	1 (1.0)	2 (0.7)	1.000
Postoperative outcomes			
Length of stay, days (median, IQR)	2 (2–3)	2 (2–3)	0.941
30-day complications, *n* (%)			
Any complication	22 (22.2)	58 (19.5)	0.661
Clavien–Dindo ≥ III	9 (9.1)	31 (10.4)	0.847
Pleural effusion requiring drainage	3 (3.0)	11 (3.7)	1.000
Pneumothorax	2 (2.0)	8 (2.7)	1.000
Reoperation	2 (2.0)	5 (1.7)	1.000
Esophageal perforation	1 (1.0)	3 (1.0)	1.000
Gastric perforation	1 (1.0)	2 (0.7)	1.000
Blood transfusion required	3 (3.0)	2 (0.7)	0.111
30-day readmission	2 (2.0)	12 (4.0)	0.530
30-day mortality	0 (0.0)	0 (0.0)	NA

*IQR* Interquartile range

### Anemia resolution

At 12-month follow-up, 92 of 99 IDA patients (93.4%) achieved anemia resolution. Mean hemoglobin increased from 10.5 ± 1.4 to 13.7 ± 1.2 g/dL at 12 months (+ 3.2 ± 1.3 g/dL, *p* < 0.001). Resolution rates by baseline severity were mild anemia 58/62 (93.5%), moderate anemia 29/31 (93.5%), and severe anemia 5/6 (83.3%). Iron supplementation was discontinued in 82 patients (82.8%) by 12 months. Resolution rate was 96 patients (97.1%) in 5 years. Control group hemoglobin remained stable (13.9 ± 1.2 to 13.9 ± 1.1 g/dL, *p* = 0.892) (Table 5).

**Table 5:** Hematologic response and anemia resolution

Parameter	Baseline	3 months	12 months	5 years	*P* value^a^
Overall hemoglobin changes (g/dL)					
Anemic group	10.5 ± 1.4	13.5 ± 1.3	13.7 ± 1.2	13.8 ± 1.1	< 0.001
Control group	13.9 ± 1.2	13.8 ± 1.1	13.9 ± 1.1	14.0 ± 1.0	0.892
Mean change in anemic group	–	+ 3.0 ± 1.2	+ 3.2 ± 1.3	+ 3.3 ± 1.2	
Hemoglobin change by baseline severity					
Mild anemia (*n* = 62)	11.2 ± 0.5	13.8 ± 1.1	14.0 ± 1.0	14.1 ± 0.9	< 0.001
Mean change	–	+ 2.6 ± 0.8	+ 2.8 ± 0.9	+ 2.9 ± 0.8	
Resolution rate, *n* (%)	–	57 (91.9)	58 (93.5)	61 (98.4)	
Moderate anemia (*n* = 31)	9.2 ± 0.6	12.9 ± 1.2	13.1 ± 1.1	13.3 ± 1.0	< 0.001
Mean change	–	+ 3.7 ± 1.0	+ 3.9 ± 1.1	+ 4.1 ± 1.1	
Resolution rate, *n* (%)	–	27 (87.1)	29 (93.5)	30 (96.8)	
Severe anemia (*n* = 6)	7.1 ± 0.7	11.8 ± 1.5	12.2 ± 1.4	12.6 ± 1.3	< 0.001
Mean change	–	+ 4.7 ± 1.2	+ 5.1 ± 1.3	+ 5.5 ± 1.2	
Resolution rate, *n* (%)	–	4 (66.7)	5 (83.3)	5 (83.3)	
Other hematologic parameters					
Ferritin (ng/mL)^b^	18.2 ± 12.3	68.4 ± 31.2	95.3 ± 38.5	102.1 ± 41.3	< 0.001
MCV (fL)	77.9 ± 9.1	86.2 ± 7.8	87.8 ± 6.9	88.1 ± 6.5	< 0.001
Transferrin saturation (%)^c^	14.2 ± 6.3	24.1 ± 7.8	26.8 ± 8.2	28.3 ± 7.9	< 0.001
CRP (mg/L)^d^	8.2 ± 6.1	3.1 ± 2.2	2.8 ± 1.9	2.6 ± 1.7	< 0.001
Iron supplementation status					
Still on iron therapy, *n* (%)	87 (87.9)	27 (27.3)	5 (5.1)	0 (0)	
Successfully discontinued, *n* (%)	–	60 (60.6)	82 (82.8)	87 (87.9)	
Overall anemia resolution					
All patients, *n* (%)	–	88 (88.9)	92 (93.4)	96 (97.1)^e^	
Quality-of-life scores^f^					
GERD-HRQL	21.3 ± 8.2	8.4 ± 5.1	6.2 ± 4.3	5.8 ± 3.9	< 0.001
MNA score	21.4 ± 1.3	23.8 ± 1.1	24.2 ± 0.9	24.3 ± 0.8	< 0.001

^a^Repeated measures ANOVA

^b^Available in 89 patients

^c^Available in 78 patients

^d^Available in 76 patients

^e^Available in 99 patients at 5 years

^f^Available in 82 patients

When hernia subtypes were analyzed separately, Type II hernias were present in 45 IDA patients (45.5%) and 140 controls (47.1%), and Type III hernias in 50 IDA patients (50.5%) and 149 controls (50.2%), with no significant difference in distribution between groups (*p* = 0.72 and *p* = 0.85, respectively). Among IDA patients, anemia resolution at 12 months was 93.3% (42/45) in Type II and 92.0% (46/50) in Type III hernias. The four patients with Type IV hernias all achieved resolution (4/4, 100%). Resolution rates did not differ significantly across hernia subtypes (*p* = 0.79), suggesting that hernia subtype classification does not independently predict anemia resolution following surgical repair (Supplementary Table 9).

### Predictors of preoperative anemia

**Fig. 3:**
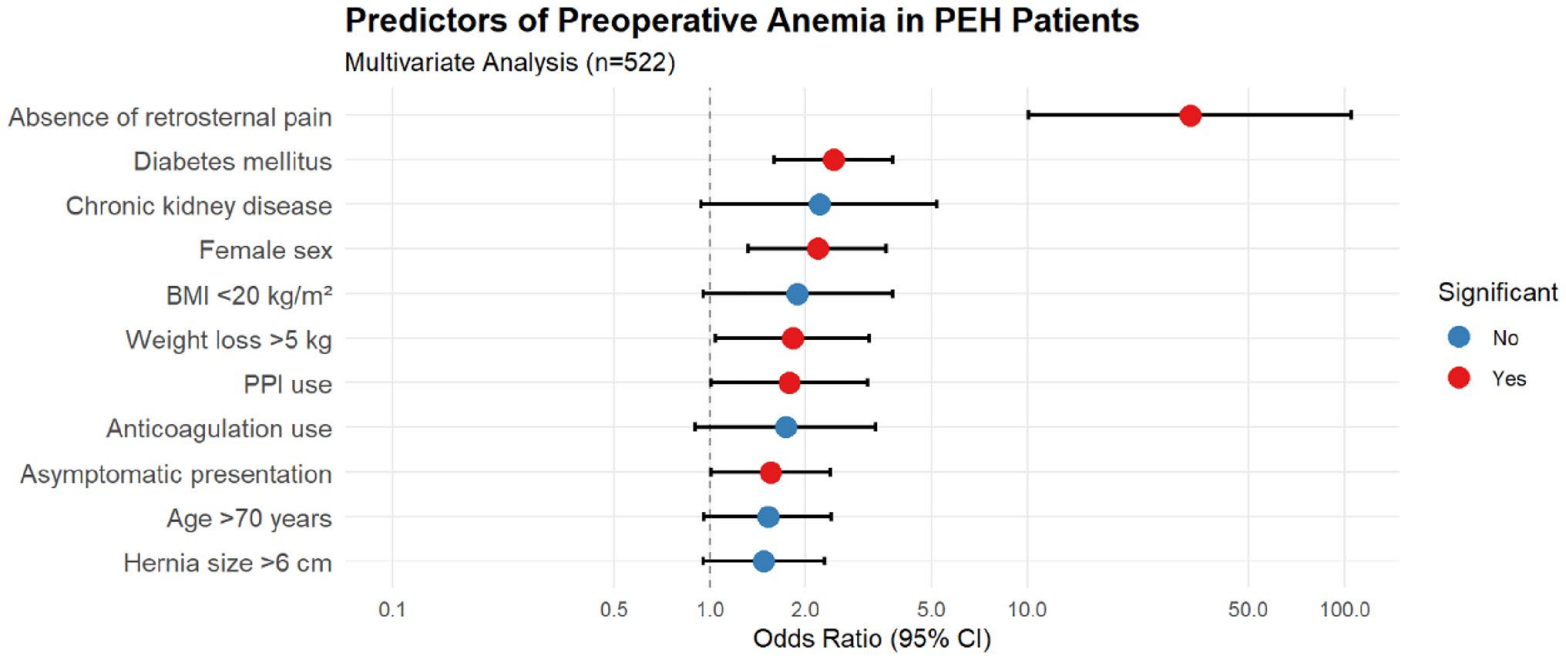
Predictors of preoperative anemia

In the unmatched cohort of 522 patients, some features functioned as Predictors of Preoperative Anemia, female sex (OR 2.18, 95% CI 1.32–3.59, *p* = 0.002), diabetes mellitus (OR 2.45, 95% CI 1.59–3.77, *p* < 0.001), and the absence of retrosternal pain (OR 32.5, 95% CI 10.1–104.6, *p* < 0.001) were the strongest predictors of preoperative anemia (Fig. 3).

### Predictors of anemia non-resolution

Seven patients (6.6%) did not achieve anemia resolution at 12 months. Due to the small number of non-resolution events, multivariate analysis was not performed. On descriptive analysis, diabetes mellitus was present in 5 of 7 non-resolvers (71.4%) compared with 26 of 92 resolvers (28.3%, *p* = 0.030), and chronic kidney disease in 2 of 7 (28.6%) versus 3 of 92 (3.3%, *p* = 0.018). Prolonged failure of iron therapy exceeding 6 months was documented in 5 non-resolvers (71.4%) versus 22 resolvers (23.9%, *p* = 0.016). Among patients with none of these three risk factors, the resolution rate was 98.1% (52/53) compared with 87.0% (40/46) in those with one or more risk factors (Table 6).

**Table 6:** Characteristics associated with anemia resolution (descriptive analysis)

Factor	Resolution (*n* = 92)	Non-resolution (*n* = 7)	*P* value^a^
Patient characteristics			
Age > 70 years	32 (34.8)	5 (71.4)	0.064
Female sex	69 (75.0)	5 (71.4)	1.000
BMI < 20 kg/m^2^	7 (7.6)	2 (28.6)	0.121
Comorbidities			
Diabetes mellitus	26 (28.3)	5 (71.4)	0.030
Chronic kidney disease	3 (3.3)	2 (28.6)	0.018
Anemia characteristics			
Severe anemia (Hb < 8 g/dL)	5 (5.4)	1 (14.3)	0.359
Baseline ferritin < 15 ng/mL^b^	48 (52.2)	1 (14.3)	0.087
Failed iron therapy > 6 months	22 (23.9)	5 (71.4)	0.016
Duration of anemia > 2 years^c^	15 (16.3)	4 (57.1)	0.028
Early response indicator			
Hemoglobin rise > 2 g/dL at 3 months	71 (77.2)	2 (28.6)	0.014
Surgical factors			
Hernia size > 6 cm	30 (32.6)	4 (57.1)	0.221
Gastropexy performed	68 (73.9)	3 (42.9)	0.163
Hernia recurrence	12 (13.0)	4 (57.1)	0.014

Values are *n* (%)

Due to the small number of non-resolution events (*n* = 7), multivariate analysis was not performed. The strongest univariate associations with non-resolution were diabetes mellitus (71.4% vs. 28.3%), chronic kidney disease (28.6% vs. 3.3%), and prolonged failed iron therapy (71.4% vs. 23.9%)

Key Finding: Among patients with none of the three major risk factors (diabetes, CKD, or failed prolonged iron therapy), resolution rate was 98.1% (52/53) compared to 87.0% (40/46) in those with ≥ 1 risk factor

^a^Fisher’s exact test used for all comparisons due to small cell counts

^b^Available in 89 patients

^c^Self-reported duration before surgery

**Table 7:** Hernia recurrence and anemia outcomes

A. Overall recurrence analysis			
Variable	IDA group (*n* = 99)	Control group (*n* = 297)	*P* value
Anatomical recurrence, *n* (%)	16 (16.2)	19 (6.4)	0.008
Time to recurrence, months	18.3 ± 12.1	22.4 ± 14.3	0.351
Size of recurrent hernia, cm^a^	3.2 ± 1.8	3.5 ± 2.1	0.642
Symptomatic recurrence, *n* (%)	7 (43.8)	12 (63.2)	0.317
Required reoperation, *n* (%)	2 (12.5)	5 (26.3)	0.414

B. Anemia outcomes in patients with recurrence (*n* = 16)			
Variable	Anemia resolved (*n* = 12)	Persistent anemia (*n* = 4)	*P* value
Age, years	61.8 ± 13.9	63.0 ± 11.5	0.874
Diabetes, *n* (%)	4 (33.3)	2 (50.0)	0.604
Baseline Hb, g/dL	9.97 ± 1.24	9.25 ± 0.42	0.283
Hb at 1 year, g/dL	14.03 ± 1.12	10.75 ± 0.50	< 0.001
Mean Hb change, g/dL	+ 4.06 ± 1.31	+ 1.50 ± 0.58	< 0.001
Size of recurrence, cm	2.9 ± 1.6	4.2 ± 2.1	0.234
Time to recurrence, months	19.2 ± 11.8	15.5 ± 10.2	0.580

75% of anemic patients maintained hemoglobin correction despite anatomical hernia recurrence, suggesting that surgical intervention may produce lasting physiological changes beyond anatomical repair

^a^Available in 28 patients

### Hernia recurrence

Mean follow-up for the entire cohort was 20.5 ± 13.4 months. Among patients completing minimum 12-month follow-up (*n* = 357; 89.9% IDA, 90.2% controls), anatomical recurrence occurred in 14 IDA patients (15.7%) versus 17 controls (6.3%, *p* = 0.006) (Table 7).

Overall, anatomical recurrence occurred in 16 IDA patients (16.2%) versus 19 controls (6.4%, *p* = 0.008), with mean time to recurrence of 18.3 ± 12.1 versus 22.4 ± 14.3 months (*p* = 0.351). Among the 16 IDA patients with recurrence, 12 (75.0%) maintained anemia resolution; mean hemoglobin increased from 9.97 ± 1.24 to 14.03 ± 1.12 g/dL (+ 4.06 ± 1.31 g/dL), compared with + 1.50 ± 0.58 g/dL in the 4 with persistent anemia (*p* < 0.001).

## Discussion

Our study demonstrates that unexplained iron deficiency anemia resolves in 93.4% of patients following paraesophageal hernia repair, with durability extending to 97.1% at 5 years. This remarkably high success rate, achieved with a mean hemoglobin increase of 3.2 g/dL (95% CI 2.9–3.5), establishes PEH repair as a highly effective treatment for this challenging clinical scenario. The consistency of our findings with the 72–95% resolution range reported in quality studies [3, 20–23], despite methodological differences, strengthens the evidence for surgical intervention.

A key methodological strength distinguishing our study from prior work, particularly the single-arm analysis by Cheverie et al. [22], is the use of propensity-matched controls. By comparing anemic patients with non-anemic patients undergoing identical procedures, we isolated the hernia–anemia relationship while controlling for surgical technique, perioperative management, and patient characteristics. The comparable postoperative outcomes between groups (Clavien–Dindo ≥ III: 9.1% vs .10.4%, *p* = 0.847) validate that surgical quality was equivalent across cohorts, may mitigate technical factors as explanations for the differential anemia response. This design provides stronger evidence that the anemia resolution is specifically attributable to the hernia repair itself rather than confounding variables.

The anemia characteristics in our cohort reveal several important paradoxes that merit discussion. Despite profound hematologic abnormalities—with 31.3% having moderate anemia and 6.1% severe anemia—42.4% of patients were completely asymptomatic at presentation. This “silent anemia” phenomenon contrasts sharply with the typical presentation of iron deficiency, where patients usually manifest fatigue, dyspnea, or other classic symptoms well before reaching such severe depletion. Furthermore, 78.2% of patients had failed to respond adequately to oral iron therapy despite a mean treatment duration of 5.8 months, suggesting that simple iron malabsorption alone cannot explain the pathophysiology. The elevated baseline CRP (8.2 ± 6.1 mg/L) that normalized postoperatively (2.6 ± 1.7 mg/L) points to an inflammatory component, while the preservation of nutritional markers (albumin 4.1 ± 0.5 g/dL, MNA score 21.4 ± 1.3) despite severe iron deficiency suggests highly selective mechanisms affecting iron metabolism rather than global malnutrition.

Critically, the normal nutritional status documented in all anemic patients (MNA scores ≥ 20) can make malnutrition and eating difficulties less probable as responsible for the observed anemia. This finding is particularly important given that dysphagia is common in PEH patients and could be associated with nutritional deficiencies. The comparable GERD-HRQL scores between anemic and control groups preoperatively (29.1 ± 3.8 vs. 28.7 ± 4.1, *p* = 0.612), including similar dysphagia subscores (1.8 ± 0.9 vs. 1.9 ± 0.8, *p* = 0.534), suggest that the anemia was not secondary to impaired oral intake or dietary restriction. These observations support a mechanism specific to the herniated stomach rather than global nutritional compromise.

Our study design systematically excluded common causes of iron deficiency anemia, creating a truly “unexplained” cohort. By design, we excluded patients with Cameron lesions, gastric ulcers, or erosive gastritis—the traditional explanations for PEH-associated anemia. Among the remaining workup, colonoscopy performed in 46.5% revealed polyps or diverticulosis in some patients, but these findings showed no correlation with anemia severity. All female patients underwent gynecologic evaluation with no cases of menometrorrhagia identified in the anemic group. Only 5.1% had chronic kidney disease, and nutritional parameters remained normal in > 90% of patients. Medication-related bleeding risks were similar between groups, with comparable rates of aspirin (17.2% vs. 11.8%) and anticoagulation use (8.1% vs. 4.0%). This comprehensive exclusion process strengthens our conclusion that the PEH itself, through mechanisms beyond visible mucosal injury, drives the anemia. It should be noted that while Cameron lesions, gastric ulcers, and erosive gastritis were excluded from the anemic cohort by study design, these findings were present in controls (17.5%, 12.8%, and 5.1%, respectively). This asymmetric exclusion was intentional to create a cohort with truly unexplained anemia, but it does represent a methodological consideration when interpreting comparisons between groups.

The resolution patterns provide insights into the pathophysiology and clinical course. The rapid response—with 88.9% achieving normal hemoglobin by 3 months—suggests that surgical correction immediately addresses the underlying mechanism. The severity-dependent recovery timeline (mild: 2.8 months, moderate: 3.9 months, severe: 5.2 months) follows expected hematopoietic physiology once the causative factor is removed. Critically, the control group showed no change in hemoglobin levels (13.9 ± 1.2 to 13.9 ± 1.1 g/dL, *p* = 0.892), suggesting that surgery specifically benefits those with pre-existing anemia rather than causing a general increase in hemoglobin. Given the very small number of transfused patients in both groups (*n* = 3 IDA, *n* = 2 controls), their inclusion or exclusion from the analysis is unlikely to have materially influenced the observed resolution rates**.** The identification of diabetes (present in 71.4% of non-responders vs. 28.3% of responders) and chronic kidney disease (28.6% vs. 3.3%) as predictors of failure suggests that systemic factors affecting erythropoiesis or iron utilization may overwhelm the benefits of anatomical repair in some patients.

The higher rate of intraoperative bleeding requiring intervention in anemic patients (10.1% vs 2.7%, *p* = 0.005) deserves particular attention. All bleeding episodes were managed laparoscopically using hemostatic agents, energy devices, or suture/clip application, with no conversions to open surgery. This finding likely indicates the presence of tissue friability secondary to chronic inflammation and nutritional microdeficiencies affecting tissue quality in patients with prolonged iron deficiency. The chronic inflammatory state suggested by elevated baseline CRP may contribute to increased tissue fragility and vascularity. Despite this technical challenge, the consistent laparoscopic management without conversions suggest that minimally invasive repair remains feasible and safe in this population. Surgeons should anticipate the possibility of increased bleeding when operating on patients with chronic unexplained anemia and ensure appropriate hemostatic resources are available.

Perhaps, our most intriguing finding is that 75% (12/16) of patients with anatomical hernia recurrence maintained normal hemoglobin levels despite failed repair. This apparent paradox has several potential explanations. Most plausibly, recurrent hernias may be smaller than the original defects—literature suggests 58.9% of recurrences measure ≤ 2 cm [24]—potentially below a threshold size for causing anemia. Alternatively, surgical intervention may produce lasting physiological changes such as improved gastric drainage, reduced inflammation, or vascular remodeling that persist despite anatomical failure. It is also possible that the initial surgery disrupted the pathological mechanism (whether chronic venous congestion, intermittent volvulus, or mucosal trauma) in a manner that remains corrected even with partial anatomical recurrence. The limitation that we did not systematically measure recurrence size prevents definitive conclusions, but this observation suggests that the relationship between anatomical defect and functional consequence is more complex than simple mechanical causation. Future studies should prospectively evaluate recurrence characteristics to better understand this phenomenon.

These findings have important clinical implications. Current SAGES guidelines recommend repair for symptomatic PEH patients and consideration for select asymptomatic patients with large hernias [14], but do not specifically address iron deficiency anemia as an independent indication. Our finding that 42.4% of anemic patients were completely asymptomatic highlights a potential gap in current algorithms. With a 93.4% cure rate balanced against a 9.1% major complication rate—consistent with the 8–12% reported by high-volume centers [6, 25–27]—the risk–benefit ratio appears favorable for patients with refractory anemia. The surgical indications in asymptomatic anemic patients in our cohort included large hernia size (> 5 cm), the presence of type III–IV hernias, and anemia refractory to medical therapy, highlighting the importance of considering surgical intervention even in the absence of traditional symptoms when significant anemia is present.

Our study has several important limitations that warrant consideration. The retrospective design precludes establishing causation, and we can only demonstrate association between surgery and anemia resolution. The high proportion of asymptomatic patients in the IDA cohort (42.4%) likely reflects referral enrichment: patients identified with unexplained IDA during hematologic workup in whom PEH was subsequently discovered as the probable etiology. While this may introduce referral bias—with a cohort that is inherently more likely to demonstrate a causal hernia–anemia relationship—it also highlights a clinically distinct and underrecognized patient subgroup in whom surgical evaluation may be warranted even in the absence of traditional foregut symptoms. The small number of non-responders (*n* = 7) prevented reliable multivariate analysis, limiting our ability to create robust predictive models. We did not perform mechanistic studies such as iron absorption testing, gastric pH measurement, or systematic inflammatory cytokine profiling that could elucidate pathophysiology. We lacked data on iron saturation and vitamin B12 levels, which could have provided additional insights into the specific mechanism of iron deficiency. Although medication-related bleeding risks were comparable between groups, the numerically higher rates of aspirin (17.2% vs. 11.8%) and anticoagulation use (8.1% vs. 4.0%) in the IDA cohort warrant acknowledgment. This study was not powered to assess the independent contribution of antithrombotic therapy to subclinical mucosal bleeding and iron loss. Prospective studies with larger sample sizes and systematic medication reconciliation would be needed to determine whether antithrombotic use modulates the anemia phenotype in patients with PEH. The single healthcare system design may limit generalizability, though our multicenter approach within that system provided standardization.

The 15-year study period (2010–2024) encompasses evolving surgical techniques, instrumentation, and perioperative care protocols, which may introduce heterogeneity. Various fundoplication techniques were employed (Nissen 360°, Toupet 270°, Dor 180°, and Thal 90°), and while outcomes appeared consistent across techniques, we cannot exclude technique-specific effects on anemia resolution. We did not systematically correlate hernia size with anemia severity or measure recurrent hernia dimensions. When CT alone did not provide adequate anatomical detail, UGI contrast series was used complementarily for hernia classification; however, no contrast-related aspiration events were recorded. Hernia size and classification were derived from formal radiologic reports generated at the time of imaging by board-certified radiologists, without independent re-reading by the surgical team for research purposes. Hernia type assignment was derived from the routine clinical documentation of the operating surgeon and reviewing radiologist, without blinded central re-adjudication. Inter-observer variability in PEH subtyping—particularly between Type II and Type III hernias—is a recognized limitation of radiologic and intraoperative classification, and we cannot quantify its magnitude within this retrospective cohort. Importantly, however, the primary clinical distinction relevant to this study—between Type I sliding hernias (excluded) and Type III–IV true paraesophageal hernias (included)—relies on anatomical features (intrathoracic stomach fraction, displaced gastroesophageal junction) that are substantially more reproducible across observers, which mitigates the likely impact on our principal findings.

Questionnaire data (MNA and GERD-HRQL) collected at variable timepoints, including retrospectively for patients operated years prior, introduce potential recall bias. Some patients were asked about their preoperative status up to 15 years after surgery, which may affect accuracy of baseline assessments. Finally, we cannot exclude the possibility that some patients had intermittent bleeding from transient Cameron lesions that healed before endoscopy.

Future research should address these limitations through prospective studies incorporating mechanistic evaluations of iron kinetics, inflammation markers, and gastric physiology before and after repair. Randomized trials comparing early surgery versus intensive medical therapy in patients with refractory anemia could establish causation. Investigation of anemia as an indication for screening for occult PEH might identify patients earlier in their disease course. Development of risk stratification tools incorporating baseline hemoglobin, ferritin levels, inflammatory markers, and the presence of diabetes or chronic kidney disease could optimize patient selection.

In conclusion, this study, as the largest to date to our knowledge, may provide evidence that paraesophageal hernia repair effectively treats unexplained iron deficiency anemia in the vast majority of patients, with durable results often maintained despite anatomical recurrence. The high prevalence of asymptomatic presentation and excellent outcomes suggest that unexplained, refractory iron deficiency anemia may warrant consideration as an indication for PEH repair in appropriately selected patients. While our retrospective findings cannot definitively establish this recommendation, they provide compelling rationale for prospective studies to evaluate whether clinical guidelines should be expanded to address this underrecognized but highly treatable condition.

## Supplementary Information

Below is the link to the electronic supplementary material.

Supplementary file1 (DOCX 446 kb)

Supplementary file2 (DOCX 14 kb)

## Declarations

### Use of Artificial Intelligence

No artificial intelligence tools were used in the data analysis, interpretation, or manuscript writing for this study.
