# Supplementary material for: Surgical repair of paraesophageal hernia resolves unexplained iron deficiency anemia in the vast majority of patients: a propensity-matched multicenter study
Source: Surg Endosc. 2026 May 21;40(6):4971–84. doi: 10.1007/s00464-026-12900-8 (PMC13246518; doi:10.1007/s00464-026-12900-8)
Supplement: Supplementary file 1 — Supplementary file1 (DOCX 446 kb) [file 464_2026_12900_MOESM1_ESM.docx]

**Supplementary Table S1. Baseline Characteristics Before Propensity Score Matching**

| **Variable** | **Total Cohort (n=552)** | **Anemic Group (n=136)** | **Control Group (n=416)** |  | **SMD** | **p-value** |
| --- | --- | --- | --- | --- | --- | --- |
| **Demographics** |  |  |  |  |  |  |
| Age, years | 61.0 ± 14.3 | 63.3 ± 14.0 | 60.4 ± 14.3 |  | 0.206 | 0.034 |
| Female sex, n (%) | 367 (66.5) | 111 (81.6) | 256 (61.5) |  | 0.305 | 0.003 |
| BMI, kg/m² | 24.4 ± 3.8 | 24.2 ± 3.3 | 24.5 ± 3.9 |  | 0.081 | 0.419 |
| **ASA score, n (%)** |  |  | 0.035 |  |  | 0.280 |
| - ASA 1 | 100 (18.1) | 23 (16.9) | 77 (18.5) |  |  |  |
| - ASA 2 | 370 (67.0) | 93 (68.4) | 277 (66.6) |  |  |  |
| - ASA 3 | 79 (14.3) | 20 (14.7) | 59 (14.2) |  |  |  |
| - ASA 4 | 3 (0.5) | 0 (0.0) | 3 (0.7) |  |  |  |
| **Comorbidities, n (%)** |  |  |  |  |  |  |
| Diabetes mellitus | 188 (34.1) | 73 (53.7) | 115 (27.6) |  | 0.569 | <0.001 |
| Hypertension | 234 (42.4) | 68 (50.0) | 166 (39.9) |  | 0.204 | 0.041 |
| Hyperlipidemia | 142 (25.7) | 41 (30.1) | 101 (24.3) |  | 0.131 | 0.181 |
| IHD | 48 (8.7) | 16 (11.8) | 32 (7.7) |  | 0.136 | 0.154 |
| COPD | 26 (4.7) | 8 (5.9) | 18 (4.3) |  | 0.072 | 0.458 |
| PPI use | 412 (74.6) | 118 (86.8) | 294 (70.7) |  | 0.397 | <0.001 |
| **Laboratory Values** |  |  |  |  |  |  |
| Hemoglobin, g/dL | 13.1 ± 1.8 | 10.5 ± 1.4 | 13.9 ± 1.2 |  | 2.613 | <0.001 |
| MCV, fL | 87.2 ± 8.1 | 78.4 ± 9.2 | 89.8 ± 6.3 |  | 1.452 | <0.001 |
| Ferritin, ng/mL* | 85.3 ± 42.1 | 18.2 ± 12.3 | 112.4 ± 38.5 |  | 3.291 | <0.001 |

*Available in 312 patients; SMD = Standardized Mean Difference; ASA = American Society of Anesthesiologists; IHD = Ischemic Heart Disease; COPD = Chronic Obstructive Pulmonary Disease; PPI = Proton Pump Inhibitor; MCV = Mean Corpuscular Volume

**Supplementary Table S2. Complete Cohort Analysis Without Matching (n=552)**

| **Variable** | **Anemic Group (n=136)** | **Control Group (n=416)** | **p-value** |
| --- | --- | --- | --- |
| **Primary Outcome** |  |  |  |
| Anemic resolution at 1 year | 127/136 (93.4%) | NA | - |
| **Secondary Outcomes** |  |  |  |
| Operative time, min | 102.3 ± 53.2 | 98.7 ± 57.1 | 0.517 |
| Blood loss, mL | 85.2 ± 62.3 | 52.3 ± 41.2 | <0.001 |
| Conversion to open, n (%) | 3 (2.2) | 5 (1.2) | 0.433 |
| Hospital stay, days | 2.8 ± 1.9 | 2.5 ± 1.6 | 0.076 |
| Overall complications, n (%) | 18 (13.2) | 42 (10.1) | 0.319 |
| Major complications, n (%) | 12 (8.8) | 28 (6.7) | 0.421 |
| Reoperation, n (%) | 5 (3.7) | 8 (1.9) | 0.326 |
| **Long-term Outcomes** |  |  |  |
| Hernia recurrence, n (%) | 20 (14.7) | 32 (7.7) | 0.018 |
| GERD-HRQL improvement* | 22.8 ± 5.1 | 23.1 ± 4.8 | 0.543 |
| PPI cessation, n (%) | 112 (82.4) | 298 (71.6) | 0.013 |

*Change from baseline; GERD-HRQL = Gastroesophageal Reflux Disease-Health Related Quality of Life

**Supplementary Table S3. Laboratory Values in Anemic Cohort**

| **Parameter** | **Baseline** | **3 Months** | **1 Year** | **5 Years** | **p-value*** |
| --- | --- | --- | --- | --- | --- |
| Hemoglobin, g/dL | 10.5 ± 1.4 | 13.5 ± 1.3 | 13.7 ± 1.2 | 13.8 ± 1.1 | <0.001 |
| MCV, fL | 78.4 ± 9.2 | 86.2 ± 7.8 | 87.8 ± 6.9 | 88.1 ± 6.5 | <0.001 |
| Ferritin, ng/mL† | 18.2 ± 12.3 | 68.4 ± 31.2 | 95.3 ± 38.5 | 102.1 ± 41.3 | <0.001 |
| CRP, mg/L‡ | - | 3.1 ± 2.2 | 2.8 ± 1.9 | 2.6 ± 1.7 | <0.001 |

*Repeated measures ANOVA; †Available in 89 patients; ‡Available in 76 patients MCV = Mean Corpuscular Volume; CRP = C-reactive Protein

**Supplementary Table S4: Comparative Analysis of Major Series on Paraesophageal Hernia Repair and Anemia Resolution**

| **Study** | **Year** | **Study Design** | **Sample Size** | **Cameron Lesions (%)** | **Anemic Anemic Resolution Rate** | **Follow-up Duration** | **Key Findings** | **Evidence Grade*** |
| --- | --- | --- | --- | --- | --- | --- | --- | --- |
| **Current Study** | 2024 | Retrospective with PSM | 136 anemic patients | 0% | 93.4% at 1 year, 97.1% at 5 years | 5 years | Resolution maintained in 80% despite hernia recurrence; inverse Cameron lesion relationship | B |
| **Cheverie et al.** [22] | 2020 | Retrospective single-arm | 56 | 32% | Overall: 46.4%; No lesions: 72.2% | Median 160 days | Patients without endoscopic lesions had better outcomes | C |
| **Carrott et al.** [3] | 2012 | Prospective observational | 270 | 23% | 83% | Mean 2 years | Anemic Anemic as common presenting symptom; high resolution with repair | B |
| **Laliberté et al.** [20] | 2021 | Retrospective | 116 | 52.6% | 95% ceased iron therapy | 1 year | Mean Hb increase: 2.5 g/dL (females), 2.5 g/dL (males) | C |
| **Hayden & Jamieson** [9] | 2005 | Retrospective | 11 | 27% | 100% | Median >2 years | Small series but complete resolution | D |
| **Panzuto et al.** [19] | 2004 | Prospective | 180 | 15.5% | 62% with surgery + PPI | 6 months | Combined medical-surgical approach | B |
| **Moskovitz et al.** | 1992 | Prospective with controls | 16 | 44% | 75% with surgery | 1 year | Early study establishing surgical benefit | C |
| **Skipworth et al.** [14] | 2014 | Prospective | 7 | 43% | 86% | 6 months | Transfusion-dependent anemic anemic resolved | D |
| **Haurani et al.** [2] | 2012 | Retrospective | 68 | 38% | 88% with lesions; 50% without | 1 year | Differential response based on endoscopy | C |

*Evidence Grade: A = Systematic review/meta-analysis; B = Well-designed cohort or case-control; C = Case series or poor-quality cohort; D = Case reports or small series

PSM = Propensity Score Matching; Hb = Hemoglobin; PPI = Proton Pump Inhibitor

**Table s5. Subgroup Analysis of Anemia Resolution by Patient Characteristics**

| **Subgroup** | **N** | **Resolution at 12 months n (%)** | **Mean Hb Change (g/dL)** | **p-value*** |
| --- | --- | --- | --- | --- |
| **Age Groups** |  |  |  | 0.031 |
| <60 years | 38 | 37 (97.4) | 3.4 ± 1.2 |  |
| 60-70 years | 33 | 31 (93.9) | 3.2 ± 1.3 |  |
| >70 years | 28 | 24 (85.7) | 2.9 ± 1.4 |  |
| **Sex** |  |  |  | 0.628 |
| Female | 74 | 69 (93.2) | 3.2 ± 1.3 |  |
| Male | 25 | 23 (92.0) | 3.1 ± 1.2 |  |
| **BMI Categories** |  |  |  | 0.042 |
| <20 kg/m² | 9 | 7 (77.8) | 2.6 ± 1.5 |  |
| 20-25 kg/m² | 52 | 49 (94.2) | 3.3 ± 1.2 |  |
| >25 kg/m² | 38 | 36 (94.7) | 3.2 ± 1.3 |  |
| **Diabetes Status** |  |  |  | 0.018 |
| No diabetes | 68 | 66 (97.1) | 3.4 ± 1.2 |  |
| Diabetes present | 31 | 26 (83.9) | 2.7 ± 1.3 |  |
| **CKD Status** |  |  |  | 0.004 |
| No CKD | 94 | 89 (94.7) | 3.3 ± 1.2 |  |
| CKD present | 5 | 3 (60.0) | 1.8 ± 1.1 |  |
| **Baseline Ferritin** |  |  |  | 0.006 |
| <15 ng/mL | 49 | 48 (98.0) | 3.6 ± 1.1 |  |
| 15-30 ng/mL | 40 | 35 (87.5) | 2.8 ± 1.3 |  |
| **Iron Therapy Duration** |  |  |  | 0.011 |
| <6 months | 65 | 63 (96.9) | 3.4 ± 1.2 |  |
| ≥6 months | 34 | 29 (85.3) | 2.8 ± 1.3 |  |
| **PPI Use** |  |  |  | 0.721 |
| No PPI | 47 | 44 (93.6) | 3.2 ± 1.3 |  |
| PPI use | 52 | 48 (92.3) | 3.1 ± 1.3 |  |

*Chi-square test for resolution rates; †Available in 89 patients

**Table s6. Subgroup Analysis by Anemia Severity and Clinical Presentation**

| **Subgroup** | **N** | **Resolution at 12 months n (%)** | **Time to Normal Hb (months)†** | **Iron Stopped n (%)** |
| --- | --- | --- | --- | --- |
| **Baseline Anemia Severity** |  |  |  |  |
| Mild (Hb 10-11.9) | 62 | 58 (93.5) | 2.8 ± 1.2 | 59 (95.2) |
| Moderate (Hb 8-9.9) | 31 | 29 (93.5) | 3.9 ± 1.8 | 29 (93.5) |
| Severe (Hb <8) | 6 | 5 (83.3) | 5.2 ± 2.1 | 6 (100.0) |
| p-value |  | 0.633 | <0.001 | 0.814 |
| **Symptom Presentation** |  |  |  |  |
| Asymptomatic | 42 | 40 (95.2) | 3.1 ± 1.4 | 41 (97.6) |
| GI symptoms only | 23 | 21 (91.3) | 3.4 ± 1.6 | 21 (91.3) |
| Anemia symptoms only | 17 | 15 (88.2) | 3.8 ± 1.9 | 16 (94.1) |
| Both GI + anemia symptoms | 17 | 16 (94.1) | 3.2 ± 1.5 | 16 (94.1) |
| p-value |  | 0.803 | 0.541 | 0.776 |
| **Retrosternal Pain** |  |  |  |  |
| Absent | 97 | 91 (93.8) | 3.3 ± 1.6 | 93 (95.9) |
| Present | 2 | 1 (50.0) | 6.0 ± 0.0 | 1 (50.0) |
| p-value |  | 0.098 | 0.042 | 0.061 |
| **Weight Loss** |  |  |  |  |
| No weight loss | 80 | 76 (95.0) | 3.2 ± 1.5 | 77 (96.3) |
| Weight loss >5kg | 19 | 16 (84.2) | 3.9 ± 1.8 | 17 (89.5) |
| p-value |  | 0.116 | 0.118 | 0.238 |

†Mean ± SD; Resolution defined as Hb >12 g/dL (females) or >13 g/dL (males) without iron supplementation

**Table s7. Subgroup Analysis by Hernia and Surgical Characteristics**

| **Subgroup** | **N** | **Resolution at 12 months n (%)** | **Recurrence n (%)** | **p-value*** |
| --- | --- | --- | --- | --- |
| **Hernia Size** |  |  |  | 0.458 |
| ≤4 cm | 21 | 20 (95.2) | 2 (9.5) |  |
| 4-6 cm | 46 | 44 (95.7) | 6 (13.0) |  |
| >6 cm | 32 | 28 (87.5) | 8 (25.0) |  |
| **Hernia Type** |  |  |  | 0.622 |
| Type II | 15 | 14 (93.3) | 2 (13.3) |  |
| Type III | 80 | 74 (92.5) | 13 (16.3) |  |
| Type IV | 4 | 4 (100.0) | 1 (25.0) |  |
| **Fundoplication Type** |  |  |  | 0.916 |
| Nissen 360° | 68 | 63 (92.6) | 11 (16.2) |  |
| Toupet 270° | 21 | 20 (95.2) | 3 (14.3) |  |
| Dor/Thal | 10 | 9 (90.0) | 2 (20.0) |  |
| **Gastropexy** |  |  |  | 0.098 |
| Not performed | 26 | 22 (84.6) | 7 (26.9) |  |
| Performed | 73 | 70 (95.9) | 9 (12.3) |  |
| **Intraoperative Bleeding** |  |  |  | 0.412 |
| No bleeding | 89 | 83 (93.3) | 14 (15.7) |  |
| Bleeding occurred | 10 | 9 (90.0) | 2 (20.0) |  |
| **Operative Time** |  |  |  | 0.241 |
| <90 minutes | 52 | 50 (96.2) | 7 (13.5) |  |
| ≥90 minutes | 47 | 42 (89.4) | 9 (19.1) |  |

*For resolution rates

**Table s8. Temporal Trends and Long-term Outcomes by Subgroups**

| **Subgroup** | **3-month Hb (g/dL)** | **1-year Hb (g/dL)** | **5-year Hb (g/dL)†** | **Sustained Resolution‡** |
| --- | --- | --- | --- | --- |
| **By Baseline Severity** |  |  |  |  |
| Mild anemia | 13.8 ± 1.1 | 14.0 ± 1.0 | 14.1 ± 0.9 | 60/62 (96.8%) |
| Moderate anemia | 12.9 ± 1.2 | 13.1 ± 1.1 | 13.3 ± 1.0 | 29/31 (93.5%) |
| Severe anemia | 11.8 ± 1.5 | 12.2 ± 1.4 | 12.6 ± 1.3 | 5/6 (83.3%) |
| **By Age Group** |  |  |  |  |
| <60 years | 13.7 ± 1.2 | 13.9 ± 1.1 | 14.0 ± 1.0 | 37/38 (97.4%) |
| 60-70 years | 13.5 ± 1.3 | 13.7 ± 1.2 | 13.8 ± 1.1 | 31/33 (93.9%) |
| >70 years | 13.2 ± 1.4 | 13.4 ± 1.3 | 13.5 ± 1.2 | 23/28 (82.1%) |
| **By Diabetes Status** |  |  |  |  |
| No diabetes | 13.6 ± 1.2 | 13.8 ± 1.1 | 13.9 ± 1.0 | 66/68 (97.1%) |
| Diabetes | 13.1 ± 1.4 | 13.3 ± 1.3 | 13.4 ± 1.2 | 25/31 (80.6%) |
| **By Recurrence Status** |  |  |  |  |
| No recurrence | 13.5 ± 1.3 | 13.7 ± 1.2 | 13.8 ± 1.1 | 80/83 (96.4%) |
| Recurrence | 13.1 ± 2.0 | 13.2 ± 1.9 | 13.4 ± 1.7 | 12/16 (75.0%) |
| **Quality of Life (GERD-HRQL)§** |  |  |  |  |
| Baseline >20 | 7.8 ± 4.9 | 5.9 ± 4.1 | 5.6 ± 3.7 | 48/52 (92.3%) |
| Baseline ≤20 | 9.2 ± 5.4 | 6.6 ± 4.5 | 6.1 ± 4.2 | 44/47 (93.6%) |

†Available in subset of patients; ‡Defined as maintaining Hb above threshold through 5 years; §Available in 82 patients

**Subgroup Analyses S5-8**

Resolution was defined as normalization of hemoglobin without need for iron supplementation or transfusion. Age-stratified resolution rates were 97.4% in patients <60 years, 93.9% in those 60–70 years, and 85.7% in those >70 years (p = 0.031). Patients without diabetes achieved higher resolution than those with diabetes (97.1% vs 83.9%, p = 0.018). Low baseline ferritin (<15 ng/mL) was associated with higher resolution compared with ferritin 15–30 ng/mL (98.0% vs 87.5%, p = 0.006). Time to hemoglobin normalization increased with anemia severity (mild 2.8 ± 1.2 months, moderate 3.9 ± 1.8 months, severe 5.2 ± 2.1 months; p < 0.001). Resolution rates did not differ significantly between asymptomatic patients and those presenting with gastrointestinal symptoms (95.2% vs 91.3%, p = 0.803). Surgical variables, including gastropexy, were not independently associated with resolution. Long-term hemoglobin levels remained stable through 5 years across all subgroups, with sustained resolution in 96.4% of patients without recurrence versus 75.0% of those with recurrence.


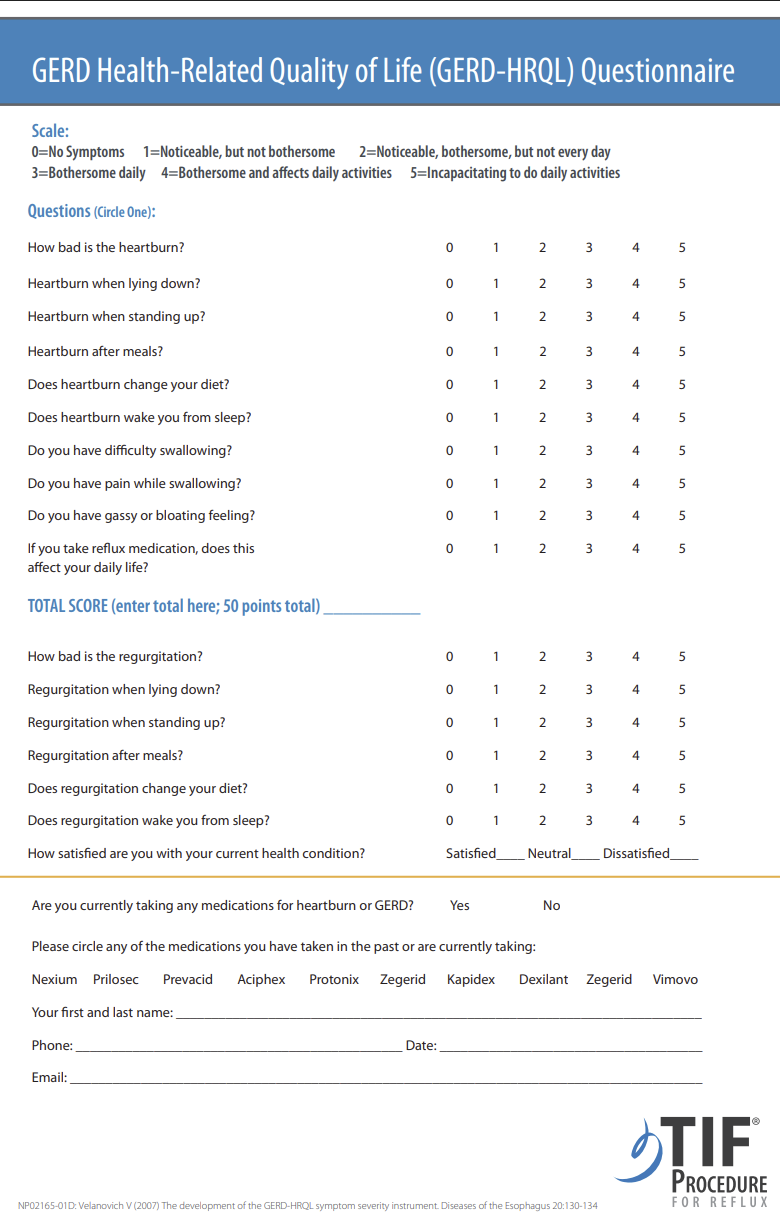


- - Velanovich V. The development of the GERD-HRQL symptom severity instrument [Internet]. 2007. Available from: <https://www.gerdhelp.com/uploads/2018/03/GERD-HRQL.pdf>


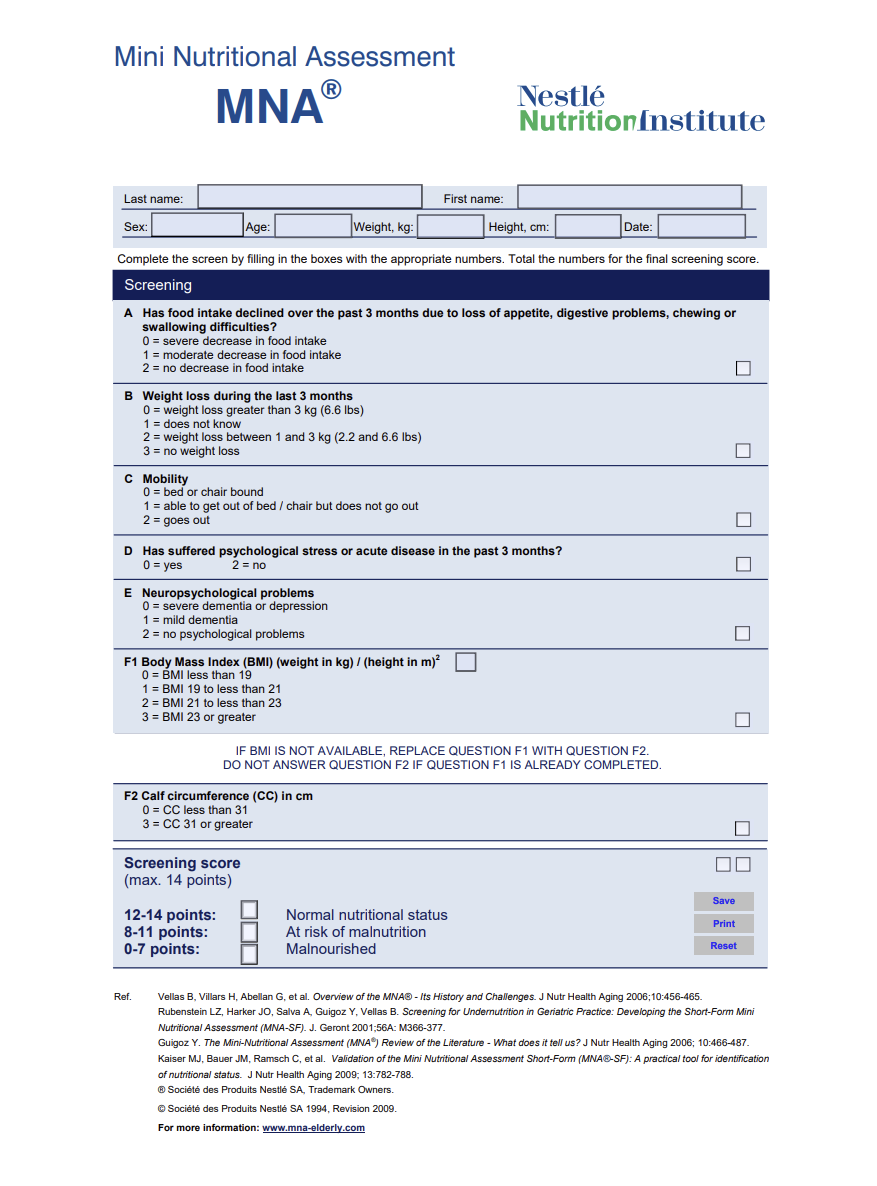
* Vellas B, Villars H, Abellan G, Soto ME, Rolland Y, Guigoz Y, Morley JE, Chumlea WC, Salva A, Rubenstein LZ, Garry PJ. Overview of the MNA® – Its history and challenges. *J Nutr Health Aging*. 2006;10(6):456–465.
