# Supplementary material for: Surgical repair of paraesophageal hernia resolves unexplained iron deficiency anemia in the vast majority of patients: a propensity-matched multicenter study
Source: Surg Endosc. 2026 May 21;40(6):4971–84. doi: 10.1007/s00464-026-12900-8 (PMC13246518; doi:10.1007/s00464-026-12900-8)
Supplement: Supplementary file 2 — Supplementary file2 (DOCX 14 kb) [file 464_2026_12900_MOESM2_ESM.docx]

**Supplementary Table 9. Hernia Classification and Anemia Resolution by Hernia Subtype in the Matched Cohort**

| **Hernia Type** | **IDA Group n (%)** | **Control Group n (%)** | **p-value*** | **Anemia Resolution in IDA n/N (%)** |
| --- | --- | --- | --- | --- |
| Type II | 45 (45.5%) | 140 (47.1%) | 0.72 | 42/45 (93.3%) |
| Type III | 50 (50.5%) | 149 (50.2%) | 0.85 | 46/50 (92.0%) |
| Type IV | 4 (4.0%) | 8 (2.7%) | 0.35 | 4/4 (100%) |
| **Total** | **99 (100%)** | **297 (100%)** | — | **92/99 (92.9%)†** |

*Chi-square or Fisher's exact test as appropriate. †Consistent with primary outcome reported in Table 4.
